# Safety and effectiveness of antiretroviral therapies for HIV-infected women and their infants and children: protocol for a systematic review and network meta-analysis

**DOI:** 10.1186/2046-4053-3-51

**Published:** 2014-05-25

**Authors:** Andrea C Tricco, Jesmin Antony, Veroniki A Angeliki, Huda Ashoor, Brian Hutton, Brenda R Hemmelgarn, David Moher, Yaron Finkelstein, Kevin Gough, Sharon E Straus

**Affiliations:** 1 Li Ka Shing Knowledge Institute, St. Michael’s Hospital, 209 Victoria Street, East Building, Toronto, ON M5B 1 T8, Canada; 2 Clinical Epidemiology Program, Centre for Practice-Changing Research, Ottawa Hospital Research Institute and University of Ottawa, 501 Smyth Road, Ottawa, ON K1H 8L6, Canada; 3 Departments of Medicine and Community Health Sciences, University of Calgary, 1403 29th St NW, Calgary, AB T2N 2T9, Canada; 4 The Hospital for Sick Children and Department of Pediatrics, University of Toronto, 555 University Avenue, Toronto, ON M5G 1X8, Canada; 5 Department of Geriatric Medicine, University of Toronto, Toronto, ON, Canada

**Keywords:** antiretroviral therapy, breastfeeding, congenital malformation, human immunodeficiency virus, fetus, mother-to-child-transmission, pregnancy

## Abstract

**Background:**

Antiretroviral therapy reduces mother-to-child transmission of human immunodeficiency virus (HIV) during pregnancy, delivery, and breastfeeding. However, these agents have been associated with preterm birth, anemia and low birth weight. We aim to evaluate the comparative safety and effectiveness of the use of antiretroviral drugs among HIV-infected women and the effects on their infants and children through a systematic review and network meta-analysis.

**Methods/Design:**

Studies examining the effects of six antiretroviral drug classes (nucleoside reverse transcriptase inhibitors, non-nucleoside reverse transcriptase inhibitors, protease inhibitors, integrase inhibitors, fusion inhibitors, co-receptor inhibitors) administered to HIV-infected pregnant women will be included. We will include randomized clinical trials (RCTs), quasi-RCTs, non-RCTs, controlled before-after, interrupted time series, cohort, registry, and case–control studies. No limitations will be imposed on publication status (that is, unpublished studies are eligible for inclusion), duration of follow-up, study conduct period, and language of dissemination. Comprehensive literature searches will be conducted in major electronic databases, including MEDLINE, EMBASE, and the Cochrane Central Register of Controlled Trials. Gray literature will be identified through searching dissertation databases, trial protocol registries, and conference abstracts.

Two team members will independently screen all citations, full-text articles, and abstract data; conflicts will be resolved through discussion. The risk of bias and methodological quality will be appraised using appropriate tools (for example, Cochrane Collaboration’s tool for assessing risk of bias, Newcastle-Ottawa Scale, and McMaster Quality Assessment Scale of Harms). If feasible and appropriate, we will conduct random effects meta-analysis. Network meta-analysis will be considered for outcomes with the greatest number of treatment comparisons available that fulfill network meta-analysis assumptions (for example, consistency of evidence between direct and indirect data, and low statistical heterogeneity between included studies).

The primary effectiveness outcome is mother-to-child transmission of HIV, and the primary safety outcome is major congenital malformation (overall and specific types) among newborns of HIV-infected women. Secondary safety outcomes include stillbirths, infant/child death, preterm delivery, overall and specific minor congenital malformations, and small for gestational age infants.

**Discussion:**

Our systematic review will be of utility to healthcare providers, policy-makers, and HIV-positive women regarding the use of antiretroviral drugs.

**Trial registration:**

PROSPERO registry number: CRD42014009071.

## Background

In 2008, more than 2.1 million children aged 15 years and younger were infected with human immunodeficiency virus (HIV) worldwide [1]. The majority of these cases were preventable [1]. Mother-to-child transmission of HIV *in-utero* and during delivery account for a large proportion of HIV infections among children born to women not being treated for HIV [2]. In addition, HIV infection can be acquired through breastfeeding of children by women who are not treated [3-5]. In fact, one randomized trial found that up to 44% of infant HIV infections were due to breastfeeding alone [6]. Other factors that increase the risk of mother-to-child transmission of HIV include delivery method (vaginal versus cesarean section), mother’s plasma RNA viral load, and gestational age [7].

In developed countries, highly active antiretroviral therapy (HAART) has reduced the mother-to-child HIV transmission rate to approximately 1 to 2% [8]. However, HAART is not available to many women living in low to middle economy countries, where other treatment regimens are routinely administered. For these women, single antiretroviral therapy may be an effective option to reduce mother-to-child transmission of HIV [9].

There are six major antiretroviral drug classes: 1) nucleoside reverse transcriptase inhibitors (NRTIs), 2) non-nucleoside reverse transcriptase inhibitors (non-NRTIs), 3) protease inhibitors, 4) integrase inhibitors, 5) fusion inhibitor, and 6) co-receptor inhibitors (also known as CCR5 antagonists) [2]. Each drug class has a unique mechanism of action and safety profile. The choice of medication regimen depends on the patient’s clinical profile (for example, pregnancy and co-infection with hepatitis B), potential adverse effects, complexity of use, availability, cost, and patient preferences.

For women who are HIV-positive, naïve to antiretroviral therapy, and pregnant, it is recommended that antiretroviral drugs are initiated after the first trimester of pregnancy [2,10,11]. This is due to increased risk of major congenital abnormalities, preterm delivery, anemia, and low birth weight [2,8,12-17]. As such, our objective is to evaluate the comparative safety and effectiveness of antiretroviral drugs in HIV-infected pregnant women and their infants who were exposed to HIV *in-utero*, during delivery or while breastfeeding, through performance of a systematic review and network meta-analysis.

## Methods/Design

### Protocol

A protocol was compiled for our systematic review and circulated for feedback from the policy-makers who posed the query within Health Canada, systematic review methodologists, clinicians, and pharmacologists. After completion, the protocol was registered with the PROSPERO database (CRD42014009071). The Preferred Reporting Items for Systematic reviews and Meta-analyses Protocols (or PRISMA-P) was used to guide the reporting of this protocol [18].

### Eligibility criteria

Only studies fulfilling our eligibility criteria will be included [19], as outlined in Additional file 1:

1. Patients: Pregnant women infected with HIV-1 and/or the fetuses and infants of mothers who are HIV-positive. Studies of women infected with HIV-2 will be excluded. Women at any stage of pregnancy will be eligible for inclusion. Infants and children exposed *in-utero*, during delivery or breastfeeding are eligible for inclusion until 18 months of age or until breastfeeding is discontinued. All countries and settings are eligible for inclusion.

2. Interventions: Any of the 24 antiretroviral medications approved for use in Canada from the aforementioned classes, as presented in Additional file 2[20]. We will include studies of all combinations and doses of these medications. In order to be included, the antiretroviral medication must be administered to HIV-infected pregnant women; we will not include studies examining antiretroviral treatment directly administered to infants or children.

3. Comparators: Antiretroviral medications against each other, against placebo/no treatment, or combinations of two or more antiretroviral drugs.

4. Outcomes: The primary effectiveness outcome is mother-to-child transmission of HIV, which is defined as infant HIV-infection from 2 weeks of age to 18 months or until breastfeeding is discontinued. The primary safety outcome is major congenital malformations (overall and by specific type), which is defined as a malformation present from birth that requires substantial medical intervention, including surgery. The secondary safety outcomes include stillbirths, infant/child death (up to 18 months of age), minor congenital malformations (overall and by specific types), small for gestational age infants (defined as weight below the 10th percentile for gestational age), and preterm delivery,

5. Study designs: Experimental (randomized clinical trials (RCTs), quasi-RCTs, and non-RCTs), quasi-experimental (controlled before and after studies and interrupted time series) and observational (cohort, case control, and registry studies) studies.

6. Other limitations: No limitations will be imposed on publication status, language of dissemination, duration of study follow-up or period of study conduct.

### Information sources and literature search

The primary source of literature will be a structured search of major electronic databases, including MEDLINE, EMBASE, and the Cochrane Central Register of Controlled Trials. The secondary source of potentially relevant material will be a search of the gray literature [21], including dissertation databases (ProQuest Dissertations and Theses Database), clinical trial registries (for example, World Health Organization International Clinical Trials Search Portal), and conference abstracts from selected international symposia on HIV. Literature saturation will be achieved by contacting antiretroviral drug manufacturers, scanning the reference lists of included studies and relevant reviews, and contacting authors who are prolific in HIV research.

The literature searches will be conducted by an experienced librarian. Our main (MEDLINE) literature search was peer-reviewed by a different librarian using the Peer Review of Electronic Search Strategies (PRESS) checklist [22]. The final MEDLINE search is provided in Additional file 3.

### Study selection process

Our draft eligibility form is presented in Additional file 1, which will be used for screening titles and abstracts (or citations) and potentially relevant full-text articles. The eligibility form will be revised, as necessary, during a calibration exercise with the team. Full screening will only occur when high agreement (for example, kappa statistic ≥60%) [23] is observed across the team. Subsequently, two team members will screen each citation and potentially relevant full-text article independently using our online screening software (synthesi.sr) [24]. Conflicts will be resolved through discussion until consensus is achieved.

### Data items and data collection process

Data will be abstracted for the following factors:

1. Study characteristics: study design, year of conduct, duration of follow-up, sample size, setting, country of study conduct, and type of antiretroviral drugs examined.

2. Patient characteristics: mean age of the mother and infant/child, gestational stage when antiretroviral medication administered, family history of congenital malformations, breastfeeding, maternal HIV viral load, maternal CD4 count, history of other sexually transmitted diseases, chorioamnionitis, prolonged rupture of membranes, mode of delivery, postpartum or intrapartum hemorrhage, history of previous stillbirth, consumption of folate, tobacco and alcohol during pregnancy.

3. Outcome results at the longest duration of follow-up (for example, congenital malformations, and infant/child mortality).

A standardized data abstraction tool will be developed in excel and calibrated with the team. Subsequently, each of the included studies will be abstracted by two team members, independently, and conflicts will be resolved through discussion. During this stage, duplicate publications (or companion reports) will be sorted [25], and authors will be contacted for data clarifications, as necessary.

### Methodological quality/risk of bias appraisal

The risk of bias of experimental and quasi-experimental studies will be appraised using the Cochrane Effective Practice and Organization of Care tool for assessing risk of bias [26]. The methodological quality of observational studies will be appraised using the Newcastle-Ottawa Scale [27]. Publication bias will be assessed using funnel plots [28]. Finally, studies reporting harms will be appraised using the McHarm tool [29].

### Synthesis of included studies

Fixed and random-effects meta-analysis will be conducted [30,31] separately for studies including patients receiving single or combinations of antiretroviral medications for RCTs. Summary estimates will be displayed along with their 95% confidence intervals (CI). Differences between fixed and random effects estimates suggest that there are differences between the estimated treatment effects of small and large studies [32]. Such differences will be examined using funnel plots and Harbord’s test for funnel plot asymmetry [33]. Funnel plots will be drawn when ten or more studies are included in the meta-analysis. Asymmetry will be explored by examining the study, clinical, and methodological characteristics of the outlier studies.

As we anticipate that most studies will be retrospective, pooled odds ratios will be calculated for the relevant outcomes, which are all dichotomous. For outcomes in which studies reported 0 events in one treatment arm, 0.5 will be added to the numerator and 1 will be added to the denominator. Studies reporting 0 events in all treatment arms for a particular outcome will be excluded from all analyses [34,35]. The placebo group will be the reference for the first meta-analysis and the network meta-analysis.

Statistical, clinical, and methodological heterogeneity will be examined prior to conducting meta-analysis. We will estimate the magnitude of statistical heterogeneity using the restricted maximum likelihood and the Q-profile method to estimate the 95% CI [36]. The proportion of variability that is due to heterogeneity rather than sampling error will also be quantified using the I^2^ measure [37]. If extensive statistical (for example, I^2^ ≥ 75%), clinical, or methodological heterogeneity [38] is observed, we will conduct meta-regression analysis. The total number of covariates examined in meta-regression will be constrained so that it is equal to 1/10 the number of studies [39]. Meta-regression analysis will explore the influence of important factors such as baseline effect sizes (source of statistical heterogeneity), gestational age when antiretroviral therapy was commenced and country/setting of study conduct (sources of clinical heterogeneity), and study quality (source of methodological heterogeneity) on the meta-analysis results. Both meta-analysis and meta-regression will be performed using the R software with the *metafor* package [40]. Missing measures of variance (for example, standard deviations and standard errors) will be imputed using established methods [41]. Sensitivity analysis will be conducted to ensure our imputations for missing data do not bias our results [42].

A random-effects network meta-analysis will be conducted to combine the different sources of evidence across a network of studies and make inferences regarding the relative effectiveness of multiple interventions [43]. Network meta-analysis will only be considered for outcomes with data on the majority of treatment comparisons, which will be examined using network diagrams. We will present the network diagrams for all outcomes to evaluate the extent to which treatments are connected. The choice of treatment nodes will be decided upon through discussions with the clinicians, methodologists, and statisticians from the team. For example, we will decide whether we will conduct a class-level versus individual drug analysis, incorporate standard/low/high dosages, and/or examine timing of administration; these decisions will be informed by a previous review of antiretroviral therapy [44].

A common estimate for the heterogeneity parameter across comparisons will be assumed and its magnitude will be judged according to the empirical heterogeneity distribution [45]. The network meta-analysis results will be presented as summary treatment effects (that is, mean difference for continuous data and odds ratios for dichotomous data) for each pair of treatments. We will perform network meta-analysis employing the methodology of multivariate meta-analysis in Stata using the *mvmeta* command [46].

Prior to embarking on network meta-analysis, we will ensure that the transitivity assumption is fulfilled, that is, the distribution of the effect modifiers is comparable across treatment comparisons [47]. For each outcome we will construct a table of important patient characteristics and draw boxplots to visually inspect the distribution (for example, age) or percentages (for example, male/female) of factors we consider as potential modifiers of the treatment effect. Lack of transitivity in a network can create statistical disagreement between direct and indirect evidence, that is, inconsistency [43,48]. Consistency between direct and indirect data will be examined locally, that is, in certain paths of the network, using the loop-specific method [49,50] and the separating indirect and direct evidence (SIDE) method [51], and *globally*, that is, evaluating the network as a whole, using the design-by-treatment interaction model (DBT) [52]. If we observe important heterogeneity and/or inconsistency, we will explore the possible sources, such as effect modifiers, as described above.

One advantage of a network meta-analysis is that it allows the ranking of the safety and effectiveness of all treatments examined. This will be conducted using ‘rankograms’, as well as the surface under the cumulative ranking curve (SUCRA) [53]. A sequential approach will be used for the network meta-analysis, first restricted to RCTs (which will be considered the primary analysis that we will base our conclusions on), second adding quasi-experimental data, and then finally incorporating data from observational studies. Such analyses will allow the determination of the contribution of non-randomized studies to the findings from RCTs.

## Discussion

Globally, more than 2 million children under the age of 15 years are infected by HIV. Most children acquire HIV from mother-to-child transmission and many of these cases are preventable. Effective antiretroviral therapy is available, yet safety concerns during pregnancy have been raised, including major congenital abnormalities, preterm delivery, anemia, and low birth weight [8,12-17]. Our findings regarding the comparative safety and effectiveness of these agents will be of great importance to policy-makers, healthcare providers, and patients. We are not aware of another systematic review including a network meta-analysis addressing this specific issue.

Numerous knowledge translation strategies will be used to ensure our results have a broad reach. For example, our proposal is an integrated knowledge translation whereby researchers work jointly with end-users (in this case, Health Canada, a regulatory agency). End-of-project knowledge translation initiatives will be conducted, including publication in peer-reviewed open access journals, conferences, dissemination meetings, policy briefs and social media.

## Abbreviations

HAART: highly active antiretroviral therapy; non-NRTIs: non-nucleoside reverse transcriptase inhibitors; NRTIs: nucleoside reverse transcriptase inhibitors; RCTs: randomized clinical trials.

## Competing interests

The authors declare that they have no competing interests.

## Authors’ contributions

ACT conceived the study, designed the study, helped obtain funding for the study, and helped write the draft protocol. JA registered the protocol with the PROSPERO database, provided input into the design, and edited the draft protocol. AV helped write the draft protocol. HA edited the draft protocol. BH, BRH, DM, YF, and KG provided input into the design, helped obtain funding for the study, and edited the draft protocol. SES conceived the study, designed the study, obtained the funding, and helped write the draft protocol. All authors read and approved the final protocol.

## Supplementary Material

Additional file 1 Draft eligibility criteria. Click here for file

Additional file 2 **List of relevant medications**[20]. Click here for file

Additional file 3 Draft MEDLINE literature search. Click here for file
