# Supplementary material for: Safety and effectiveness of antiretroviral therapies for HIV-infected women and their infants and children: protocol for a systematic review and network meta-analysis
Source: Syst Rev. 2014 May 25;3:51. doi: 10.1186/2046-4053-3-51 (PMC4039063; doi:10.1186/2046-4053-3-51)
Supplement: Additional file 1 — Draft eligibility criteria. [file 2046-4053-3-51-S1.doc]

**Additional file 1: Draft eligibility criteria**

**Level 1 screening**

1. Does the study include pregnant women with HIV-1 and/or their fetuses/children?

YES____ NO____ UNCLEAR____

1. Were the HIV-infected pregnant women treated with antiretroviral medication? Both monotherapy and combination therapy will be included. YES____ NO____ UNCLEAR____
2. Are participants treated with antiretroviral drugs compared to placebo/no treatment or each other? YES____ NO____ UNCLEAR____
3. Is this a relevant study design (e.g., experimental, quasi-experimental or observational)? YES____ NO____ UNCLEAR____

If you answer NO to any of these questions, the citation will be excluded. All other citations will be included.

**Level 2 screening**

1. Does the study include pregnant women with HIV-1 and/or their fetuses/children that were exposed to antiretroviral drugs *in-utero*, delivery or during breastfeeding? YES____ NO____ UNCLEAR____
2. Were the HIV-infected pregnant women treated with antiretroviral medication? Both monotherapy and combination therapy will be included. YES____ NO____ UNCLEAR____
3. Are participants treated with antiretroviral drugs compared to placebo/no treatment or each other? YES____ NO____ UNCLEAR____
4. Does the study report at least one of our outcomes of interest (e.g., major congenital malformation, mother-to-child transmission, small for gestational age infants, preterm delivery, stillbirths)? YES____ NO____ UNCLEAR____
5. Is this a relevant study design (e.g., experimental, quasi-experimental or observational)? YES____ NO____ UNCLEAR____

If you answer NO to any of these questions, the citation/study will be excluded. All other full-text articles will be included.
