# Supplementary material for: Safety and effectiveness of antiretroviral therapies for HIV-infected women and their infants and children: protocol for a systematic review and network meta-analysis
Source: Syst Rev. 2014 May 25;3:51. doi: 10.1186/2046-4053-3-51 (PMC4039063; doi:10.1186/2046-4053-3-51)
Supplement: Additional file 2 — List of relevant medications[20]. [file 2046-4053-3-51-S2.doc]

**Additional file 2: List of relevant medications**

| **Common name** | **Brand name** |
| --- | --- |
| ***Nukes (Nucleoside Reverse Transcriptase Inhibitors [NRTIs])*** | |
| abacavir, ABC | Ziagen |
| didanosine, ddI | Videx EC (enteric-coated) |
| emtricitabine, FTC | FTC is only available in Canada co-formulated with tenofovir, as Truvada |
| lamivudine, 3TC | 3TC |
| stavudine, d4T | Zerit |
| tenofovir | Viread |
| zidovudine, AZT | Retrovir |
| ***Non-nukes [NNRTIs]*** | |
| delaviridine | Rescriptor |
| efavirenz | Sustiva |
| etravirine | Intelence |
| nevirapine | Viramune |
| rilpivirine | Edurant |
| ***Protease inhibitors*** | |
| atazanavir | Reyataz |
| darunavir | Prezista |
| fosamprenavir | Telzir |
| indinavir | Crixivan |
| lopinavir | Kaletra |
| nelfinavir | Viracept |
| ritonavir | Norvir |
| saquinavir | Invirase |
| tipranavir | Aptivus |
| ***Integrase inhibitor*** | |
| raltegravir | Isentress |
| ***Fusion inhibitors*** | |
| enfuvirtide, T-20 | Fuzeon |
| ***Co-receptor inhibitor/CCR5 antagonist*** | |
| maraviroc | Celsentri |
| We will also **include** co-formulations. Co-formulations take two or more different drugs and combine them into a single pill. The following are some **examples**: | |
| **Co-formulation** | **Brand name** |
| efavirenz + FTC + tenofovir | [Atripla](http://www.catie.ca/fact-sheets/co-formulations/atripla) |
| AZT + 3TC | [Combivir](http://www.catie.ca/fact-sheets/co-formulations/combivir) |
| tenofovir + FTC + rilpivirine | [Complera](http://www.catie.ca/en/fact-sheets/co-formulations/complera) |
| lopinavir + ritonavir | [Kaletra](http://www.catie.ca/fact-sheets/protease-inhibitors/kaletra-lopinavirritonavir) |
| 3TC + abacavir | [Kivexa](http://www.catie.ca/fact-sheets/co-formulations/kivexa) |
| AZT + 3TC + abacavir | [Trizivir](http://www.catie.ca/fact-sheets/co-formulations/trizivir) |
| FTC + tenofovir | [Truvada](http://www.catie.ca/fact-sheets/co-formulations/truvada) |
